# Blood transcriptomic signatures link β-amyloid deposition to molecular pathways across SCD, MCI, and dementia

**DOI:** 10.3389/fnagi.2026.1816733

**Published:** 2026-07-15

**Authors:** Yijia Lin, Lizhen Cheng, Zhen Zhang, Liang Cui, Wei Li, Qihao Guo, Ya Miao

**Affiliations:** Department of Geriatrics, Shanghai Sixth People's Hospital Affiliated to Shanghai Jiao Tong University School of Medicine, Shanghai, China

**Keywords:** Aβ-PET, cognitive decline, magnetic resonance imaging, peripheral blood transcriptomics, RNA-Seq

## Abstract

**Introduction:**

Brain β-amyloid (Aβ) accumulation is a hallmark of Alzheimer’s disease, but noninvasive detection remains challenging. Blood transcriptomics may provide accessible biomarkers associated with Aβ pathology.

**Methods:**

We integrated peripheral blood transcriptomic profiling and MRI-derived structural metrics from 48 individuals across the cognitive continuum, including subjective cognitive decline (SCD), mild cognitive impairment (MCI), and dementia, stratified by Aβ-PET status. Predictive models were constructed using leave-one-out cross-validation (LOOCV), and selected genes were validated by qRT-PCR.

**Results:**

Cross-stage analysis identified *RUNX1T1* and *COL14A1* as consistently downregulated in Aβ-positive individuals regardless of clinical stage. A predictive model incorporating these two genes demonstrated moderate discrimination of Aβ-PET status in internal leave-one-out cross-validation (LOOCV) evaluation (AUC = 0.81). In addition, *COL14A1* expression was associated with cortical thickness and hippocampal volume, whereas *RUNX1T1* was primarily associated with hippocampal structure. Among individuals with MCI or dementia, *HCN1* and *NRG3* were upregulated, whereas *KCNMB2* was downregulated in Aβ-positive subjects. A three-gene model based on these markers achieved an LOOCV AUC of 0.79.

**Discussion:**

These findings indicate that peripheral blood transcriptomic alterations are associated with Aβ pathology across clinical groups. *RUNX1T1* and *COL14A1* represent candidate peripheral biomarkers associated with Aβ pathology, while the observed molecular signatures and their associations with brain structural measures provide a basis for further validation in larger, longitudinal cohorts and mechanistic studies.

## 1 Introduction

Accumulation of β-amyloid (Aβ) in the brain is a hallmark of cognitive decline and contributes to progressive neurodegenerative changes. According to the amyloid cascade hypothesis, Aβ is produced via sequential cleavage of amyloid precursor protein (APP) by β- and *γ*-secretases. Aggregation of the Aβ42 isoform forms extracellular plaques that impair synaptic transmission, induce oxidative stress, and trigger neuroinflammation (Jack et al., 2010; Selkoe and Hardy, 2016). Concurrently, tau hyperphosphorylation leads to neurofibrillary tangles (NFTs), closely associated with neuronal loss and cognitive decline (He et al., 2018). Increasing evidence suggests that while Aβ accumulation may initiate the disease process, tau pathology more directly reflects neurodegeneration and clinical progression, and the interaction between Aβ and tau jointly drives disease evolution.

Clinically, Aβ deposition is primarily detected through Aβ-PET imaging and cerebrospinal fluid (CSF) analysis (Mastenbroek et al., 2024). While Aβ-PET provides high sensitivity (92%) and specificity (95%) (Sabri et al., 2015), its high cost and limited accessibility restrict its widespread clinical use. CSF analysis, though more affordable, is invasive and unsuitable for routine screening, highlighting the need for non-invasive and cost-effective alternatives.

Our previous meta-analysis confirmed that plasma Aβ42 levels [95% Confidence Interval (CI) − 0.80 to −0.41, *p* < 0.0001] and the plasma Aβ42/Aβ40 ratio (95% CI − 2.17 to −0.72, *p* < 0.0001) differ significantly between Aβ-PET negative and positive patients (Cheng et al., 2022). Blood-based biomarkers have been explored as a promising solution, but several challenges remain. Plasma Aβ oligomers are present in extremely low concentrations and tend to bind strongly to albumin, complicating accurate measurement (Kulenkampff et al., 2021). Moreover, the plasma Aβ42/Aβ40 ratio varies considerably across detection methods and demonstrates only moderate predictive efficacy for Aβ-PET status [area under the curve (AUC) = 0.668] (Park et al., 2017). Recent studies have shown that the plasma APP_669–711_/Aβ_1-42_ ratio is closely associated with cognitive function in Alzheimer’s disease (AD) patients and holds potential as an early biomarker for detecting Aβ pathology (Noguchi-Shinohara et al., 2025).

Beyond Aβ-based plasma markers, recent advances in blood-based biomarkers have highlighted phosphorylated tau species, particularly p-tau217, as highly sensitive indicators of AD pathology. In recent years, plasma phosphorylated tau species, particularly p-tau217, have demonstrated excellent diagnostic performance for identifying AD pathology and show strong concordance with Aβ-PET and tau-PET imaging (Barthélemy et al., 2024; Mattsson-Carlgren et al., 2020; Pilotto et al., 2025). These markers represent a major advance in blood-based diagnosis of AD.

However, plasma tau biomarkers primarily function as highly sensitive diagnostic readouts and provide limited insight into the broader molecular mechanisms and biological pathways underlying Aβ-associated neurodegeneration. In contrast, transcriptomic profiling captures coordinated gene expression changes across immune regulation, extracellular matrix remodeling, synaptic function, and metabolic pathways, thereby offering a systems-level view of disease biology rather than a single-pathology indicator.

Furthermore, neuroimaging studies suggest that Aβ deposition is accompanied by structural alterations in cortical and hippocampal regions, which may serve as intermediate phenotypes linking peripheral molecular changes to central pathology (Salman et al., 2024). Integrating transcriptomic signatures with magnetic resonance imaging (MRI)–derived brain structure metrics could enhance the biological interpretability and predictive power of blood-based markers.

Importantly, SCD—a stage preceding mild cognitive impairment (MCI)—represents a crucial window for early intervention. Individuals with SCD often show subtle cognitive complaints without measurable deficits, yet increasing evidence suggests that a subset already exhibits abnormal Aβ deposition and early neurodegenerative changes (Shao et al., 2024; Li et al., 2022). Therefore, identifying molecular alterations in SCD may help capture the earliest biological signals of Alzheimer’s pathology before irreversible neuronal damage occurs.

Extending blood-based biomarker research, this study linked peripheral gene expression with cerebral Aβ deposition. Participants were categorized into six groups by combining cognitive status (SCD, MCI, and dementia) with Aβ-PET status. This design enabled us to capture both stage-specific and shared transcriptomic alterations associated with Aβ accumulation and to explore the relationship between blood gene expression and brain structural changes. Our findings provide new insights into the peripheral molecular correlates of Aβ pathology and support the potential of blood transcriptomics as a minimally invasive, cost-effective tool for early detection of Aβ-associated cognitive impairment processes.

## 2 Methods

### 2.1 Recruitment of participants

Participants were recruited between October 2023 and October 2024 from the Geriatrics Department of Shanghai Sixth People’s Hospital and community hospitals in Shanghai. Informed consent was obtained from all participants or their legal representatives. The study was approved by the institutional ethics committees of Shanghai Sixth People’s Hospital, affiliated with Shanghai Jiao Tong University.

### 2.2 Neuropsychology

A comprehensive neuropsychological assessment was conducted for all participants, adapted for the Chinese population. Global cognition was measured using the Mini-Mental State Examination (MMSE) (Katzman et al., 1988) and Montreal Cognitive Assessment-Basic (MoCA-BC) (Chen et al., 2016), while memory, language, and executive function were evaluated with the Auditory Verbal Learning Test (AVLT) (Zhao et al., 2015), Animal Fluency Test (AFT) (Zhao et al., 2013a) and the Boston Naming Test (BNT) (Williams et al., 1989), and Shape Trail Test (STT) (Zhao et al., 2013b).

### 2.3 Diagnostic criteria

SCD: Individuals with unimpaired cognition and who met the following criteria were identified as SCD (Jessen et al., 2014; Jack et al., 2018): a self-experienced persistent decline in cognitive capacity (decline in memory domain must be included), compared with a previously normal cognitive status, which is unrelated to an acute event.

MCI: Diagnosed using the actuarial neuropsychological method proposed by Jak and Bondi, requiring (1) impairment (>1 SD below the age-adjusted normative mean) in at least two tests within the same cognitive domain, and (2) impairment in at least one test across all three cognitive domains, without meeting the diagnostic criteria for dementia (First et al., 2023). Based on Aβ-PET results, MCI was further classified as MCI (+) and MCI (−).

Dementia: Diagnosed according to the DSM-5 criteria (First et al., 2023). Etiological classification within the dementia group was based on established guidelines: dementia was defined following the 2011 National Institute on Aging and Alzheimer’s Association (NIA-AA) criteria, combined with a syndromic dementia diagnosis and Aβ-PET positivity (Jack et al., 2018). Based on Aβ-PET results, dementia was further classified as dementia (+) and dementia (−).

### 2.4 Exclusion criteria

Participants were excluded if they had severe psychiatric disorders, substance abuse, neurological or systemic diseases affecting cognition (e.g., stroke, cancer, thyroid dysfunction), sensory impairments, a history of cognition-altering medications, contrast agent allergies, or any condition preventing completion of cognitive assessments or PET scans.

### 2.5 Aβ-PET imaging and analysis

Aβ-PET imaging was performed using ^18F-florbetapir (AV45). All scans were acquired on a Siemens Biograph mCT PET/CT scanner (Siemens Healthineers, Erlangen, Germany). Each participant received an intravenous injection of 370 MBq (10 mCi) of tracer, followed by a 50-min uptake period and a 20-min emission scan.

Images were reconstructed using filtered back projection (FBP) with attenuation correction and co-registered with CT images. PET preprocessing and quantitative analyses were performed using MATLAB (MathWorks Inc., Natick, MA, USA) and SPM12 (Wellcome Centre for Human Neuroimaging, UCL, UK).

Partial volume effect (PVE) correction was applied. Standardized uptake value ratios (SUVRs) were calculated using the cerebellar cortex as reference. Regional SUVR values were extracted by registering PET images to individual T1-weighted MRI space and parcellating them using the Automated Anatomical Labeling (AAL) atlas (90 regions).

Aβ-PET status was determined using both visual assessment and quantitative SUVR analysis. Visual interpretation was performed independently by two experienced nuclear medicine physicians, with disagreements resolved by a senior reader. Aβ positivity on visual assessment was defined as cortical tracer uptake equal to or greater than that of the adjacent white matter. A quantitative threshold of SUVR ≥ 1.5 was used for classification, based on previous longitudinal PiB-PET studies that validated this cutoff for distinguishing individuals with higher versus lower amyloid burden (Villemagne et al., 2013). To improve classification specificity and minimize potential misclassification of borderline cases, participants were classified as Aβ-PET positive only when both visual assessment and quantitative SUVR analysis were concordantly positive. Cases with discordant visual and quantitative classifications were excluded from the final analysis cohort.

### 2.6 MRI acquisition

Structural MRI was acquired on a 3.0 T Siemens Prisma scanner. High-resolution T1-weighted images were obtained using a 3D MP-RAGE sequence (TR = 3,000 ms, TE = 2.56 ms, TI = 900 ms, flip angle = 7°, FOV = 256 × 256 mm^2^, voxel size = 0.8 × 0.8 × 0.8 mm^3^).

### 2.7 Structural MRI processing and feature extraction

Structural MRI data were processed using FreeSurfer version 7.3.2^1^ via the standard recon-all pipeline. High-resolution T1-weighted images were first visually inspected to exclude scans with severe motion artifacts. The remaining images underwent automated preprocessing, including image registration, skull stripping, brain tissue segmentation, tessellation of the gray–white matter boundary, topology correction, and surface deformation. Cortical thickness measures were derived from the surface-based reconstruction and cortical parcellation outputs generated by recon-all.

Hippocampal and hippocampal subfield volumes were obtained separately using the automated hippocampal subfield segmentation module implemented in FreeSurfer. This procedure generated bilateral hippocampal subfield volumes, including CA1, CA2/3, CA4, molecular layer, granule cell layer of the dentate gyrus, hippocampal tail, subiculum, presubiculum, parasubiculum, fimbria, hippocampal fissure, and hippocampal–amygdala transition area. Volumetric measures were corrected for total intracranial volume to account for individual differences in head size. All FreeSurfer outputs, including cortical reconstruction and hippocampal subfield segmentation results, underwent visual quality control. Scans with severe motion artifacts, reconstruction errors, or unreliable segmentation were excluded before statistical analysis.

### 2.8 Imaging quality control and final sample sizes

All imaging data underwent systematic quality control before statistical analysis. PET images were evaluated for reconstruction quality and spatial registration accuracy. Structural MRI data were assessed for motion artifacts, reconstruction errors, segmentation reliability, registration accuracy, and data completeness. Scans that failed to meet quality-control criteria were excluded from further analysis.

For the Aβ-PET group-based structural MRI comparisons, 48 participants were initially included in the imaging cohort. Because segmentation success and data completeness varied across regions of interest and imaging-derived measures, the final sample size differed slightly across analyses. Specifically, cortical volume and cortical thickness analyses included 17 Aβ-PET-positive and 19 Aβ-PET-negative participants, whereas hippocampal subfield analyses included 18 Aβ-PET-positive and 22 Aβ-PET-negative participants.

For MRI–molecular correlation analyses, 30 participants with available molecular profiling data, including RNA-seq or PCR validation data, were initially eligible. After additional quality control of MRI data and exclusion of participants with registration failure, unreliable segmentation, or incomplete imaging data, 20 participants were included in the final MRI–molecular correlation analyses.

### 2.9 Blood sample collection and processing

Peripheral blood (5 mL) was collected, centrifuged at 2,000 rpm for 25 min, and plasma was removed. Residual red blood cells were lysed (#G2015, Servicebio, China), and the pellet was treated with TRIzol Reagent (G3013, Servicebio, China) for RNA preservation. Samples were stored at −80 °C.

### 2.10 RNA-seq and data preprocessing

Total RNA was isolated from PBMCs using TRIzol® Reagent (#15596026CN, Invitrogen, USA) following the manufacturer’s instructions, with removal of genomic DNA contamination. RNA-seq libraries were prepared from 1 μg total RNA using the TruSeq™ RNA Sample Prep Kit (Illumina, USA). Poly(A) + mRNA was enriched with oligo(dT) beads, fragmented, and then reverse-transcribed to cDNA. Libraries were size-selected (200–300 bp), quantified by TBS380 fluorometer, and sequenced on the Illumina HiSeq platform with paired-end 2 × 151 bp reads.

Raw reads were processed with Trimmomatic for quality control by trimming adapters, removing low-quality bases (Q < 20), reads containing >10% ambiguous bases (Ns), or shorter than 70 bp. Clean reads were aligned to the human reference genome (GRCh38) using HISAT2. Gene-level read counts were generated using featureCounts. Differential expression analysis was performed using the edgeR package with group status as the primary variable of interest. Prior to downstream analysis, baseline characteristics including age, sex, years of education, and APOE ε4 carrier status were compared across groups (Table 1), and no statistically significant differences were observed. Therefore, these variables were not included as covariates in the differential expression model. Potential batch effects arising from library preparation and sequencing were minimized during data preprocessing and quality control. Genes with |log_2_ fold change (log_2_ FC)| > 1 and adjusted *p*-value < 0.05 (Benjamini–Hochberg correction) were considered significantly differentially expressed.

**Table 1:** Baseline Characteristics of the participants.

Characteristic	SCD			MCI			Dementia		
	PET (−) (*n* = 8)	PET (+) (*n* = 8)	*P* value	PET (−) (*n* = 8)	PET (+) (*n* = 8)	*P* value	PET (−) (*n* = 8)	PET (+) (*n* = 8)	*P* value
Age	66.90 ± 6.47	65.00 ± 5.88	0.792	71.00 ± 2.38	71.60 ± 6.65	>0.999	75.90 ± 4.76	74 ± 6.48	0.982
Female (%)	75	37.5	0.3147	50	75	0.6084	50	37.5	>0.999
education (y)	15.38 ± 4.41	11.25 ± 1.98	0.737	10.38 ± 3.58	11.63 ± 2.72	0.919	10.88 ± 4.19	9.13 ± 5.17	0.939
APOE ɛ4 carriers	1/8	1/8	>0.999	0/8	3/8	0.200	1/8	3/8	0.569
MMSE	27.63 ± 1.69	27.75 ± 1.91	>0.999	27.75 ± 1.49	26.13 ± 1.25	0.9398	20.88 ± 3.00	16.88 ± 5.03	0.056
MoCA-B	25.25 ± 1.83	25.88 ± 2.30	>0.999	22.00 ± 2.78	20.25 ± 3.33	0.990	14.75 ± 5.09	12.43 ± 5.26	0.825
AVLT delayed recall	3.88 ± 2.75	4.56 ± 2.38	>0.999	1.75 ± 1.16	1.19 ± 1.46	0.982	2.20 ± 2.02	2.13 ± 4.25	>0.999
AVLT recognition	12.13 ± 8.51	18.63 ± 7.61	0.0972	15.25 ± 6.39	14.13 ± 7.59	>0.999	5.10 ± 5.62	11.25 ± 2.06	0.690
AFT	15.63 ± 6.23	15.13 ± 3.18	0.6489	14.25 ± 2.05	15.13 ± 3.36	>0.999	10.13 ± 6.17	9.43 ± 1.40	0.998
BNT	24.5 ± 2.98	24.38 ± 3.02	>0.999	22.25 ± 2.96	21.00 ± 6.23	>0.999	19.67 ± 6.41	20.50 ± 3.11	>0.999
STT-A	41.88 ± 11.01	41.25 ± 13.14	>0.999	55.25 ± 12.49	60.38 ± 19.59	0.972	63.60 ± 19.36	79.00 ± 24.70	0.733
STT-B	125.75 ± 28.98	103.00 ± 27.6	0.9618	155.00 ± 37.04	167.38 ± 47.76	>0.999	228.60 ± 62.64	225.25 ± 102.36	>0.999

SCD, subjective cognitive decline; MCI, mild cognitive impairment; APOE, apolipoprotein; MMSE, Mini-Mental State Examination; MoCA-BC, Chinese version of Montreal Cognitive Assessment-Basic; ACE-III-CV, Chinese version of Addenbrooke’s Cognitive Examination III; AVLT, Auditory Verbal Learning Test; AFT, Animal Verbal Fluency Test; BNT, Boston Naming Test; STT-A and B, Shape Trail Test Part A and B; Aβ, amyloid-β; FA, formic acid.

### 2.11 Differential expression analysis and functional enrichment

Differentially expressed genes (DEGs) were identified using a threshold of |log_2_FC| > 1 and adjusted *p* < 0.05. For multiple datasets, the Robust Rank Aggregation (RRA) method combined ranked gene lists, with aggregated p < 0.05 considered significant. Functional enrichment of DEGs was performed using Gene Ontology (GO) and Kyoto Encyclopedia of Genes and Genomes (KEGG) analyses via clusterProfiler. Over-representation analysis (hypergeometric test) with Benjamini-Hochberg correction was applied, and terms/pathways with adjusted *p* < 0.05 were considered significant.

### 2.12 xCell-based immune deconvolution analysis

Peripheral blood immune cell composition was estimated using the xCell algorithm based on bulk transcriptomic data. Gene expression matrices were input into xCell to infer relative enrichment scores for 64 immune and stromal cell types. To assess potential confounding effects of cellular heterogeneity, estimated cell-type scores were compared between Aβ-PET(+) and Aβ-PET(−) groups. Statistical differences were evaluated using the Wilcoxon rank-sum test, and *p* values were adjusted using the Benjamini–Hochberg false discovery rate (FDR) method. Cell types with adjusted *p* < 0.05 were considered statistically significant.

### 2.13 Protein–protein interaction analysis

The protein–protein interaction (PPI) network was constructed using the STRING database with a high-confidence interaction score threshold of 0.9 to ensure reliable interactions. Core genes and sub-networks within the network were identified using Cytoscape based on the maximal clique centrality (MCC) method. The top 10 core genes were selected.

### 2.14 Quantitative RT–qPCR

Total RNA was extracted using the RNAsimple Total RNA Kit (#DP419, TIANGEN, Beijing, China) according to the manufacturer’s instructions. Complementary DNA (cDNA) was synthesized using the All-in-one 5 × RT Master Mix kit (#G592, ABM, Canada) following the recommended protocol. Subsequently, qRT-PCR was performed with Blastaq™ 2 × qPCR MasterMix (#G891, ABM, Canada). The raw Ct values were collected, and relative mRNA expression levels were calculated. Primer sequences used are listed in Table S1.

For downstream validation and predictive modeling, five samples were randomly selected from each clinical subgroup within the qRT-PCR cohort to construct balanced analytical datasets.

### 2.15 LASSO logistic regression

LASSO logistic regression was performed using the R package glmnet to identify gene signatures associated with Aβ-PET positivity. The optimal regularization parameter (*λ*) was initially determined by 10-fold cross-validation within the training procedure. Genes with non-zero coefficients at the optimal λ were retained in the final models.

Given the limited sample size of the study cohort, model performance was further evaluated using leave-one-out cross-validation (LOOCV), which provides a less biased estimate of predictive accuracy in small datasets. For each LOOCV iteration, one sample was held out for testing while the remaining samples were used for model fitting, and this procedure was repeated until every sample had served as the test case once.

Receiver operating characteristic (ROC) curves were generated based on both the apparent model predictions and the LOOCV-derived predictions. The AUC was used to assess model discrimination. Because of the exploratory nature of the study and the lack of an independent external validation cohort, all predictive performance estimates should be considered internally validated results.

Candidate genes included in the LASSO models were selected prior to LOOCV using the full dataset based on differential expression analysis and RRA integration. Therefore, the reported LOOCV performance should be interpreted as a post-selection internal validation estimate rather than a fully nested cross-validation or independent validation of biomarker performance.

### 2.16 Statistical methods

All data are expressed as mean ± standard error of the mean (SEM). Continuous variables were analyzed using Student’s t-test for two-group comparisons or one-way analysis of variance (ANOVA) followed by Fisher’s Least Significant Difference (LSD) *post hoc* test for multiple group comparisons. Categorical variables were analyzed using the χ^2^ test. Spearman correlation analysis was performed to assess associations between PBMC gene expression levels and MRI-derived brain structural measures, including cortical thickness and hippocampal volume.

To control for multiple comparisons in MRI analyses, the Benjamini–Hochberg FDR correction was applied to regional group comparisons and gene–MRI correlation analyses involving multiple brain regions. Adjusted *p* values (FDR-corrected p values, q values) < 0.05 were considered statistically significant.

All statistical analyses were performed using GraphPad Prism 10.0. All tests were two-tailed. For analyses without multiple comparisons, a *p*-value < 0.05 was considered statistically significant. For MRI regional group comparisons and gene–MRI correlation analyses involving multiple testing, FDR-adjusted *p* values (q values) < 0.05 were considered statistically significant. Statisticians were blinded to group allocation during analysis.

## 3 Results

### 3.1 Participant characteristics

This study included a total of 48 participants, categorized into six groups based on cognitive status and Aβ-PET results: SCD (−), SCD (+), MCI (−), MCI (+), dementia (−), and dementia (+), with 8 individuals in each group. Detailed characteristics are presented in Table 1.

Demographic and cognitive characteristics were generally comparable between Aβ-PET positive and negative subgroups within each cognitive category. There were no significant differences in age, sex distribution, or years of education across groups. Although the proportion of APOE ɛ4 carriers tended to be higher in Aβ-PET positive individuals within the MCI and dementia categories, the differences did not reach statistical significance.

Cognitive performance, as measured by MMSE, MoCA-BC, and episodic memory tests (AVLT-delayed recall, AVLT-recognition), showed no significant differences between PET subgroups across all diagnostic categories. Similarly, executive function and language abilities, assessed by AFT, BNT, STT-A, and STT-B, did not differ significantly. Overall, the baseline characteristics were well balanced between PET-positive and PET-negative groups, minimizing the potential for confounding effects in subsequent analyses.

### 3.2 Cortical and hippocampal structural alterations associated with aβ-PET positivity

We compared MRI-derived structural metrics between all Aβ-PET positive and negative individuals across the three diagnostic stages. The results revealed a general trend toward cortical atrophy in the Aβ-PET positive group, predominantly involving reductions in global cortical volume ratio, particularly in key regions such as the precuneus and superior frontal cortex (Supplementary Figure S1A, Supplementary Table S2). In addition, significant volume reductions were observed in multiple hippocampal subfields—including CA1, CA3, CA4, dentate gyrus, subiculum, and the whole hippocampus—among Aβ-PET positive individuals compared to their PET-negative counterparts (Supplementary Figures S1B,C). Overall, Aβ-PET positivity was closely associated with widespread structural atrophy in both hippocampal and cortical regions.

### 3.3 Global transcriptomic alterations associated with aβ accumulation across cognitive states

**Figure 1:**
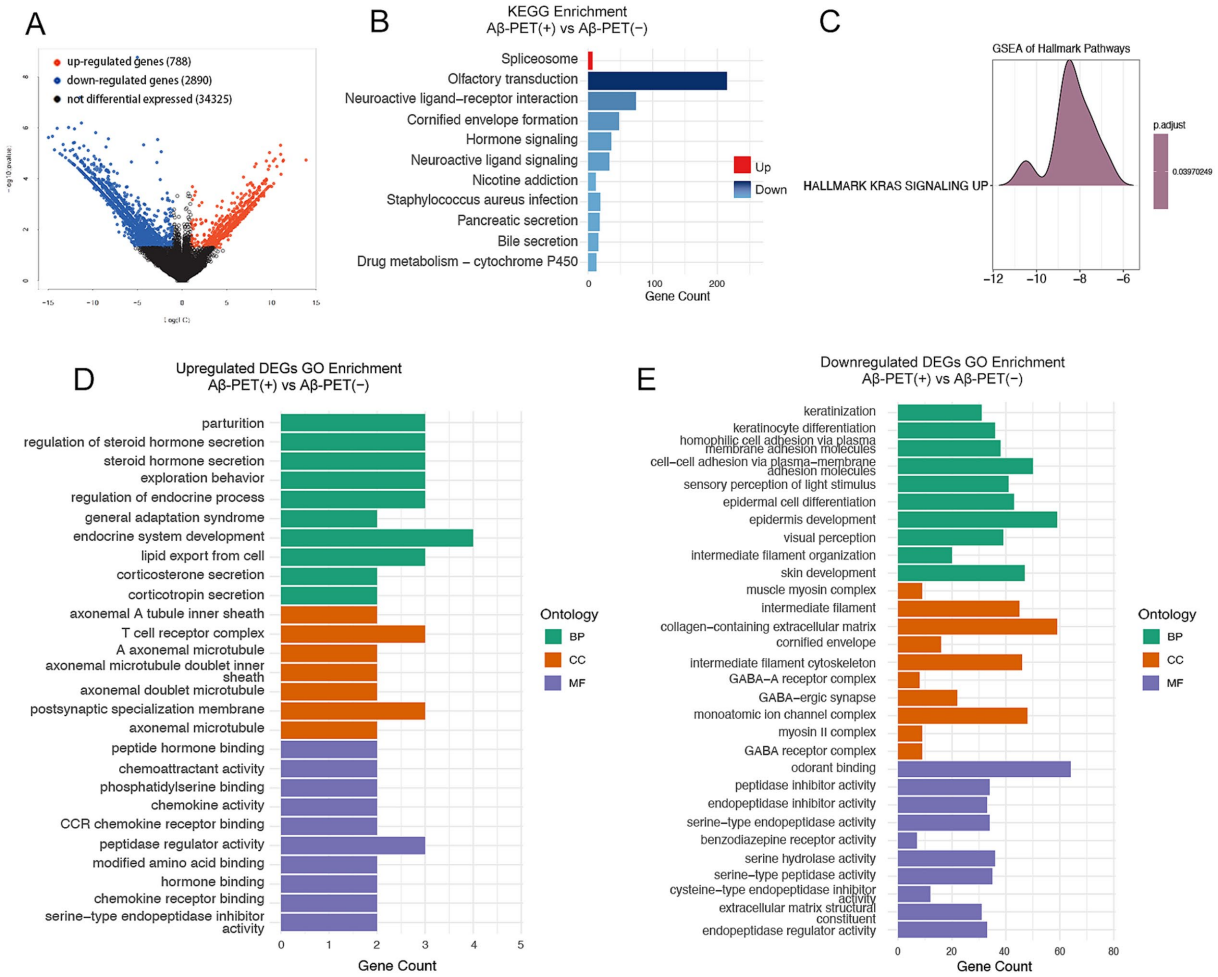
Functional enrichment analysis of differentially expressed genes (DEGs) between Aβ-PET(+) and Aβ-PET(−) individuals. **(A)** Volcano plots; **(B)** KEGG pathway enrichment; **(C)** GSEA of Hallmark pathways; **(D)** Gene Ontology (GO) enrichment of upregulated DEGs; **(E)** GO enrichment of downregulated DEGs.

To systematically elucidate transcriptomic alterations associated with cerebral Aβ accumulation, we first performed a global analysis comparing Aβ-PET(+) individuals with Aβ-PET(−) counterparts across all cognitive states (|log₂FC| > 1, adjusted *p* < 0.05). This analysis identified 788 upregulated and 2,890 downregulated genes (Figure 1A), indicating that Aβ accumulation is associated with widespread transcriptional repression rather than isolated gene-level perturbations.

Functional enrichment analysis revealed that downregulated genes were predominantly associated with protease regulation, extracellular matrix (ECM) and cytoskeletal organization, cornified envelope formation, and GABA receptor complex–related pathways (Figure 1D). These alterations suggest that Aβ deposition may broadly disrupt structural support systems, inhibitory neurotransmission, and barrier-related functions, thereby reducing neuronal stability and network resilience at a systems level. In parallel, enrichment of downregulated pathways related to ECM and cytoskeletal organization highlights a potential link between Aβ accumulation and impaired cell–matrix interactions, which may predispose neural tissue to synaptic and vascular vulnerability.

Conversely, upregulated genes were enriched in protease inhibitor activity, chemokine receptor binding, hormone binding and secretion, endocrine system development, exploratory behavior, as well as postsynaptic specialization and microtubule-related functions (Figure 1E). This transcriptional profile likely reflects a combination of compensatory and stress-induced responses, whereby immune–endocrine signaling and synaptic remodeling pathways are transiently activated in an attempt to counteract Aβ-related injury and maintain neural plasticity.

KEGG pathway analysis further demonstrated that downregulated genes were primarily involved in drug metabolism, bile and pancreatic secretion, immune responses, and neuroactive ligand–receptor interactions, whereas upregulated genes were mainly enriched in spliceosome-related pathways (Figure 1B). Together, these findings suggest that Aβ pathology is associated with broad peripheral molecular alterations across cognitive states, including pathways related to metabolic regulation, immune function, and post-transcriptional processes.

Consistently, GSEA of Hallmark pathways identified significant downregulation of KRAS signaling (NES = −1.88, q = 0.028; Figure 1C), suggesting a global suppression of intracellular signaling and metabolic activity associated with Aβ burden. Taken together, these global transcriptomic alterations support a model in which cerebral Aβ accumulation induces a coordinated shift toward structural destabilization, impaired neurotransmission, and dysregulated compensatory signaling, providing a molecular framework for subsequent stage-specific transcriptomic divergence observed across the continuum from SCD to MCI and dementia with Aβ pathology.

Considering the sensitivity of peripheral blood transcriptomic profiles to inter-individual variation in immune cell composition, we performed xCell-based deconvolution analysis to assess potential cellular confounding. Group comparisons using the Wilcoxon rank-sum test showed no significant differences in major immune cell populations between Aβ-PET(+) and Aβ-PET(−) groups, including monocytes (*p* = 0.31), B cells (*p* = 0.09), CD8 + T cells (*p* = 0.75), and NK cells (*p* = 0.69) (Supplementary Figure S2). These results suggest that the observed transcriptomic alterations are unlikely to be driven by differences in blood cell composition.

### 3.4 Stage-specific transcriptomic changes associated with aβ deposition

**Figure 2:**
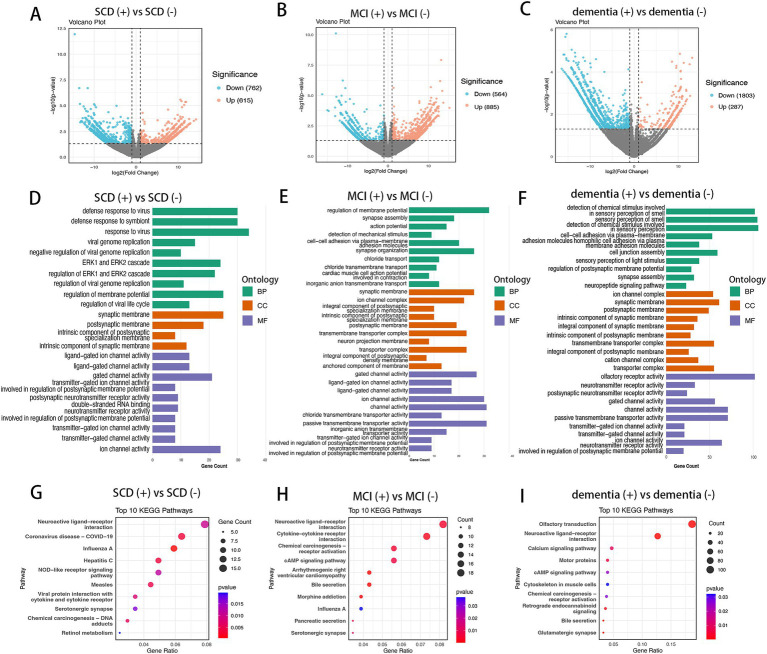
Transcriptomic profiling in SCD, MCI, and dementia groups based on Aβ-PET status**. (A–C)** Volcano plots showing differentially expressed genes (DEGs) between Aβ-PET positive and negative individuals in the SCD **(A)**, MCI **(B)**, and dementia **(C)** groups. Each point represents a gene; red dots indicate significantly upregulated genes, blue dots indicate significantly downregulated genes, and gray dots indicate non-significant genes. Significance was defined as adjusted *p* < 0.05 and |log2 FC| > 1. **(D–F)** Gene Ontology (GO) enrichment analysis of DEGs in the SCD **(D)**, MCI **(E)**, and dementia **(F)** groups. Bar plots display the top 10 significantly enriched terms in three GO categories: biological process (BP), cellular component (CC), and molecular function (MF). Only terms with adjusted p < 0.05 was included. **(G–I)** KEGG pathway enrichment analysis of DEGs in the SCD **(G)**, MCI **(H)**, and dementia **(I)** groups. The top 10 pathways most significantly enriched in each group are shown (adjusted p < 0.05).

To explore stage-specific transcriptomic changes associated with Aβ deposition, subgroup analyses were conducted in individuals with SCD, MCI, and dementia by comparing Aβ-PET(+) and Aβ-PET(−) cases (|log₂FC| > 1, adjusted *p* < 0.05). In the SCD, MCI, and dementia groups, 615, 885, and 287 genes were upregulated, while 762, 564, and 1,803 genes were downregulated, respectively (Figures 2A–C), indicating distinct transcriptional response patterns across cognitive stages.

Functional enrichment analyses revealed both shared and stage-dependent features. Across all three groups, enriched pathways consistently involved synaptic membrane organization, ion channel activity, and neurotransmitter receptor function, including ligand-gated and voltage-gated ion channels, highlighting synaptic transmission and regulation of neuronal excitability as core molecular features associated with Aβ pathology irrespective of cognitive stage.

In the SCD group, enriched pathways were predominantly related to antiviral defense programs and MAPK/ERK signaling, indicating engagement of immune-related and stress-responsive signaling pathways at the earliest stage of Aβ accumulation. In the MCI group, enriched pathways shifted toward membrane potential regulation, synapse assembly, and cell adhesion, with prominent involvement of chloride transport and ligand-gated ion channels, suggesting pronounced alterations in synaptic organization and electrophysiological properties during this intermediate stage.

In contrast, the dementia group exhibited enrichment of pathways associated with neuropeptide signaling and sensory processing, including olfactory and photoreception-related processes, reflecting broader disruption of neural communication systems at advanced stages (Figures 2G–I). KEGG pathway analysis further demonstrated consistent enrichment of neuroactive ligand–receptor interaction across all stages, whereas stage-specific enrichment of metabolic, endocrine, and signaling pathways underscored the differential functional impact of Aβ accumulation across cognitive states (Figures 2D–F).

### 3.5 Screening of hub genes in the PPI network

STRING network analysis of differentially expressed genes between Aβ-PET positive and negative individuals revealed key PPI modules (Supplementary Figure S3). Based on the STRING database and Cytoscape software, PPI networks were constructed for DEGs identified between Aβ-PET(+) and Aβ-PET(−) individuals within each cognitive category: SCD, MCI, and dementia. The top 10 hub genes were identified for each group.

**Figure 3:**
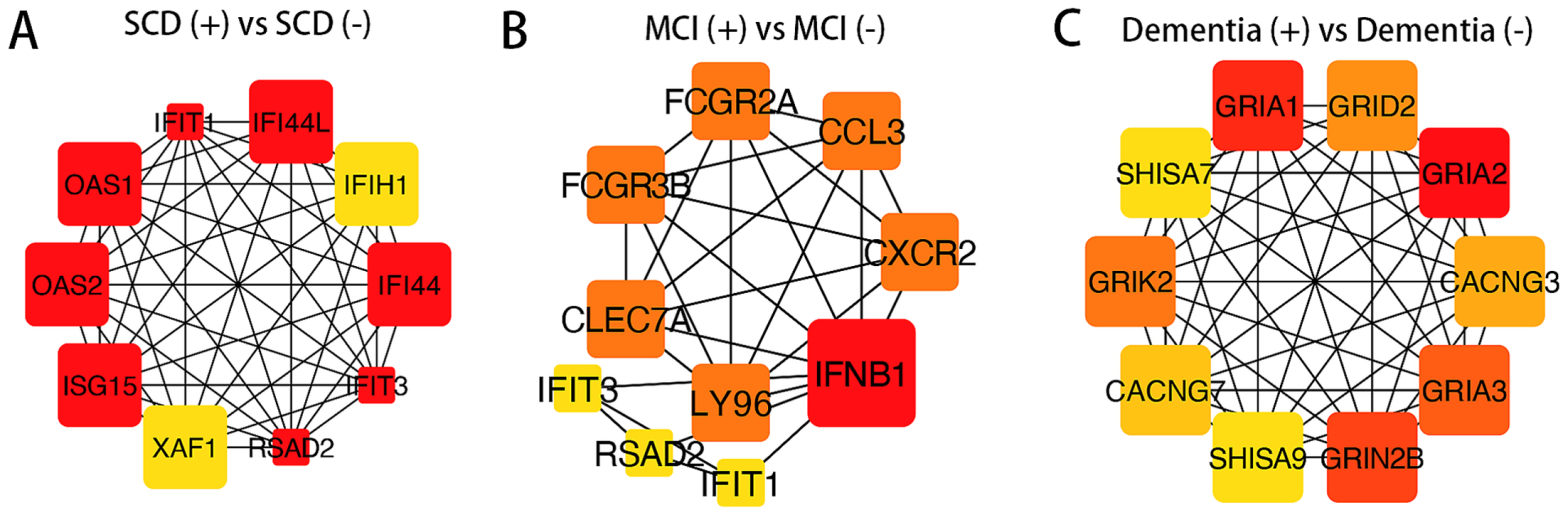
Networks of Top 10 hub genes between Aβ-PET(+) and Aβ-PET(−). **(A)** SCD group; **(B)** MCI group; **(C)** Dementia group. Only DEGs with adjusted p < 0.05 and |log₂FC| > 1 were included. The top 10 hub genes were identified using the maximal clique centrality (MCC) algorithm in the Cytoscape CytoHubba plugin. Node size reflects degree of connectivity; edge thickness represents interaction strength.

In the SCD group, hub genes were mainly associated with interferon signaling (e.g., *IFI44*, *IFI44L*, and *IFIT1*) and antiviral immune responses (e.g., *RSAD2* and *IFIT1*) (Figure 3A). In the MCI category, hub genes—identified through comparison of MCI (+) and MCI (−)—largely overlapped with those in the SCD group and were enriched in immune response and inflammation-related pathways (*RSAD2*), as well as antiviral responses (e.g., *IFIT1* and *IFIT3*) (Figure 3B). This continuity indicates that immune activation represents a sustained molecular feature during the early and intermediate stages of Aβ-associated pathology.

In contrast, the dementia group displayed a distinct PPI network architecture, with hub genes primarily involved in glutamatergic synaptic signaling (e.g., *GRIA1* and *GRIA2*) and ion channel activity (e.g., *CACNG3* and *GRIA2*) (Figure 3C). This shift suggests that, as the disease progresses, transcriptomic alterations transition from immune-dominated responses toward pathways directly related to synaptic transmission and neuronal excitability, reflecting progressive synaptic dysfunction and neural network breakdown.

Collectively, these stage-dependent PPI patterns support a dynamic model in which early Aβ accumulation is associated with prominent antiviral and innate immune activation during the SCD and MCI stages, whereas advanced disease is characterized by disruption of synaptic and ion channel–related networks, consistent with irreversible neuronal dysfunction in dementia.

### 3.6 Identification of common DEGs across cognitive categories

**Figure 4:**
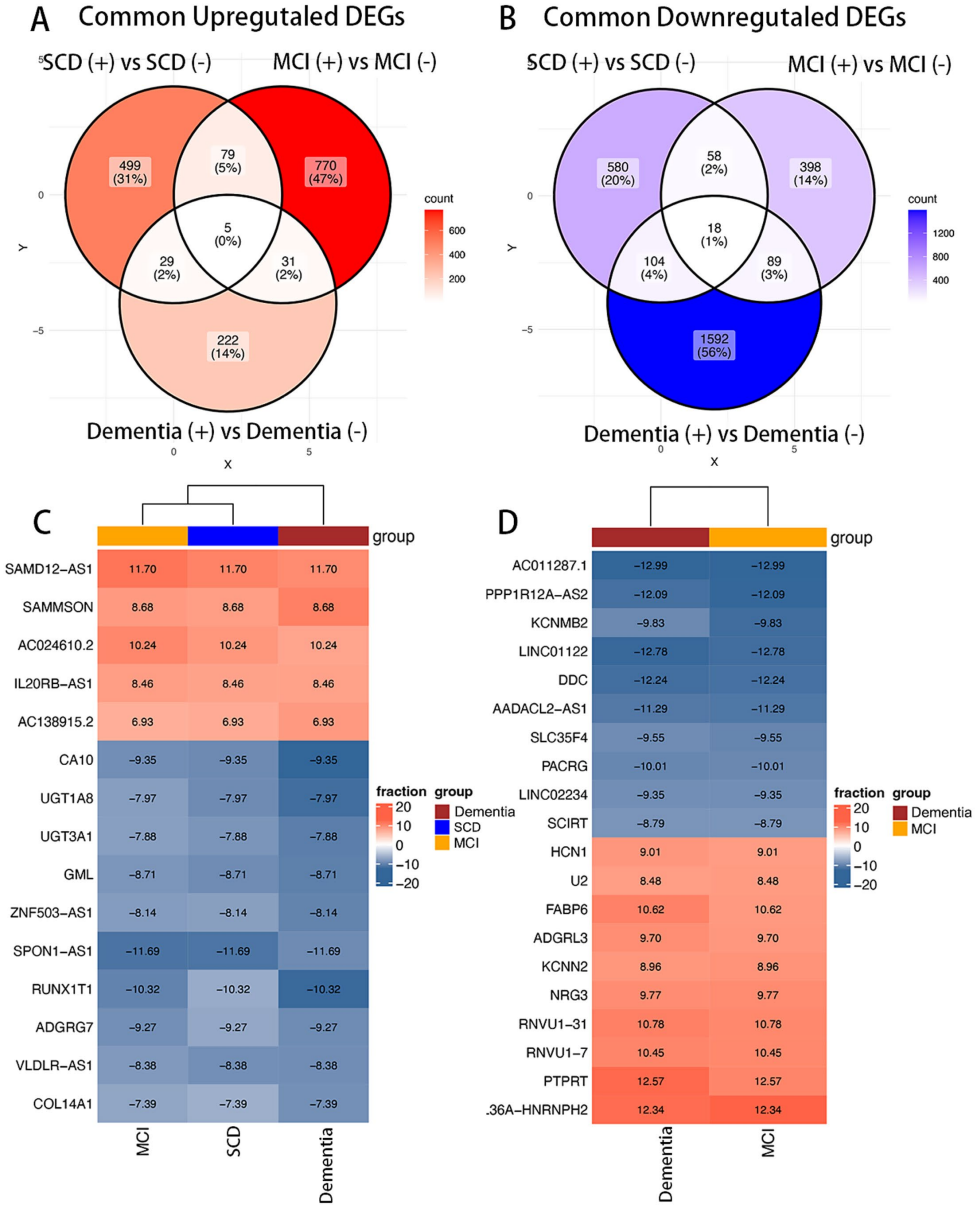
Identification of common DEGs across SCD, MCI, and dementia based on Aβ-PET status. **(A,B)** Venn diagram illustrating the number of genes commonly upregulated **(A)** and downregulated **(B)** in Aβ-PET positive (+) versus Aβ-PET negative (−) individuals across SCD, MCI, and dementia groups. **(C)** Heatmap depicting expression levels of the top 5 upregulated (all available) and top 10 downregulated genes commonly dysregulated across all three stages. Genes are ranked by Robust Rank Aggregation (RRA) scores. **(D)** Heatmap depicting expression patterns of the top 10 upregulated and top 10 downregulated genes shared between MCI and dementia groups.

To identify transcriptomic alterations that are consistently associated with Aβ-PET status independent of cognitive stage, we integrated differential expression results from Aβ-PET(+) versus Aβ-PET(−) comparisons across the SCD, MCI, and dementia groups. Genes that were uniformly upregulated or downregulated in all three categories were defined as common DEGs and visualized using Venn diagrams, revealing 5 commonly upregulated (Figure 4A) and 18 commonly downregulated genes (Figure 4B). These genes likely represent trait-like molecular signatures associated with cerebral Aβ deposition rather than stage-specific effects. To prioritize the most robust candidates, the RRA method was applied, and the top 5 upregulated (all available) and top 10 downregulated genes were visualized in a heatmap (Figure 4C).

In parallel, we investigated genes that were consistently dysregulated in both the MCI and dementia groups but not in the SCD group by intersecting DEGs from MCI Aβ-PET(+) vs. MCI Aβ-PET(−) and dementia Aβ-PET(+) vs. dementia Aβ-PET(−) comparisons. This analysis identified 31 commonly upregulated (Figure 4A) and 89 commonly downregulated genes (Figure 4B). Unlike the cross-stage common DEGs, these genes may represent molecular features associated with Aβ pathology in the MCI and dementia groups. Functional enrichment patterns suggested differences in biological processes represented in these later clinical stages compared with those observed in SCD. RRA analysis was likewise applied to this subset to identify the most consistently ranked genes, with the top 10 upregulated and top 10 downregulated genes displayed in Figure 4D.

### 3.7 Validation of hub genes

To validate the RNA-seq results, we selected mRNA transcripts for qRT-PCR analysis, excluding non-coding RNAs such as snoRNA, lncRNA, and miRNA, as they do not directly encode proteins and have distinct biological functions unrelated to protein-coding regulatory pathways.

Based on RRA analysis comparing Aβ-PET(+) to Aβ-PET(−) across the SCD, MCI, and dementia groups, all five commonly upregulated genes were found to be non-coding RNAs and thus excluded from qRT-PCR validation. Among the top ten downregulated genes ranked by RRA, non-coding RNAs were excluded, and six protein-coding genes that were highly ranked and previously reported to be associated with neurodegenerative disease pathology (Ma et al., 2020; Bergmann et al., 2022; Sun et al., 2020; Salluzzo et al., 2023; Chik et al., 2022; Zou et al., 2019). These included *COL14A1*, *RUNX1T1*, *UGT1A8*, *GML*, *UGT3A1*, and *CA10*, and were subsequently validated using qRT-PCR (Supplementary Figures S4A–F). Among these candidates, *RUNX1T1* and *COL14A1* showed expression patterns consistent with the RNA-seq results, exhibiting concordant downregulation in Aβ-PET(+) compared with Aβ-PET(−) individuals across the SCD, MCI, and dementia groups (Figure S4A–B, *p* < 0.001). These findings suggest that *RUNX1T1* and *COL14A1* may represent candidate peripheral biomarkers associated with Aβ pathology and warrant further investigation in larger independent cohorts.

In addition, we validated genes that were differentially expressed between Aβ-PET(+) and Aβ-PET(−) individuals specifically in the MCI and dementia groups, but not in the SCD group. These genes were selected based on their significant differential expression in MCI and dementia groups and previous literature reporting their association with neurodegenerative diseases (Zhang et al., 2024; Wang et al., 2014; Liu et al., 2023; Dugan et al., 2021; Del Campo et al., 2023; Taylor et al., 2009). We selected three upregulated genes (*HCN1*, *NRG3*, and *PTPRT*) and three downregulated genes (*KCNMB2*, DDC, and *PACRG*) for qRT-PCR validation. Among them, *HCN1, NRG3*, and *KCNMB2* exhibited expression trends consistent with the transcriptomic data (Supplementary Figures S4G–L), suggesting their potential relevance to stage-associated molecular alterations related to Aβ pathology.

### 3.8 Blood-based gene signatures predictive of aβ deposition across cognitive stages

To identify potential blood-based biomarkers predictive of Aβ deposition, we first performed LASSO logistic regression using the six commonly downregulated protein-coding genes identified across all cognitive stages (SCD, MCI, and dementia). The final model retained *COL14A1* and *RUNX1T1* as key predictors, with coefficients of −2.729 and −4.262, respectively, indicating a strong negative association with Aβ-PET positivity.

**Figure 5:**
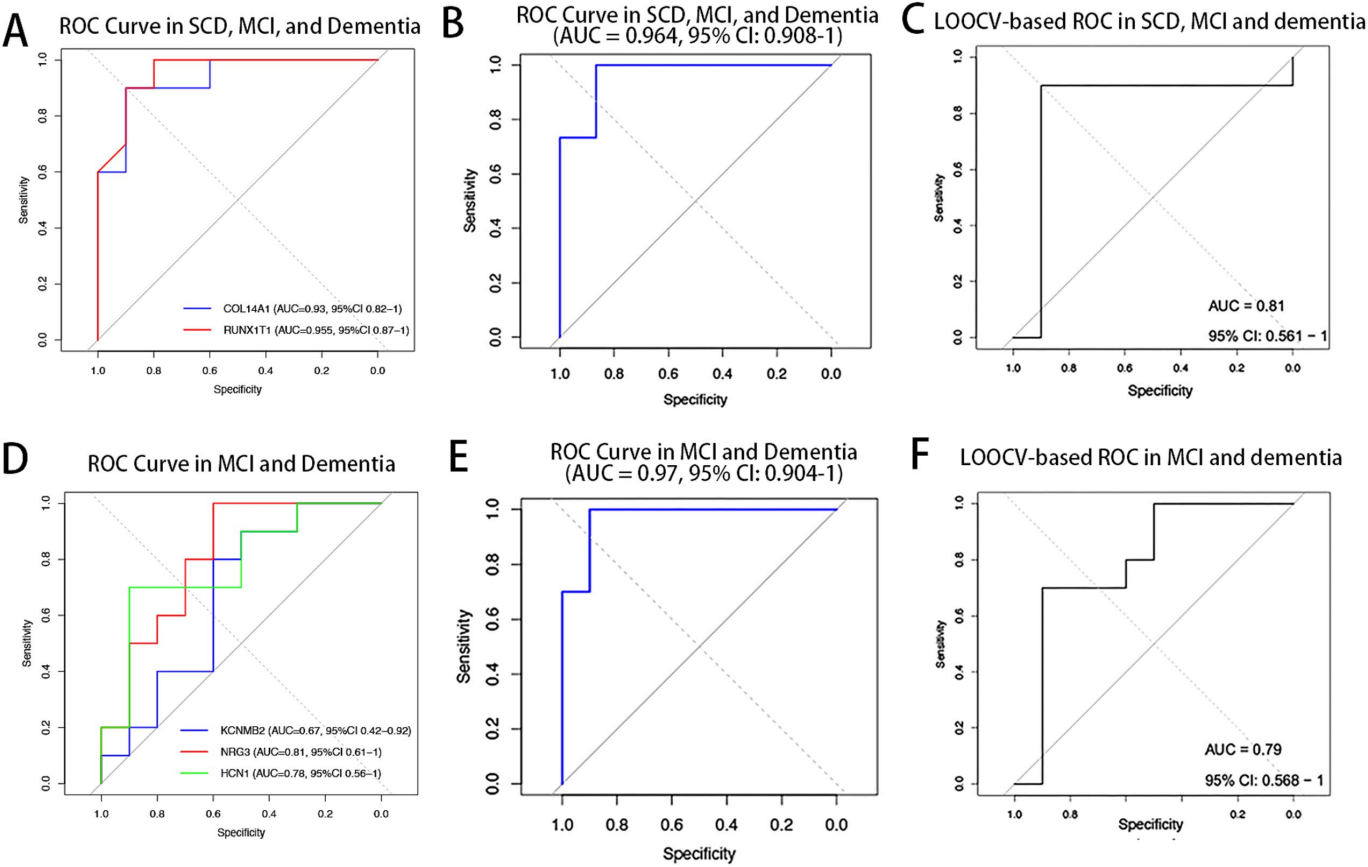
LASSO logistic regression analysis for predicting Aβ-PET status. **(A)** AUC for the LASSO model including *COL14A1* and *RUNX1T1* across all groups (SCD, MCI, dementia); **(B)** ROC curve of the two-gene model (*COL14A1* and *RUNX1T1*) distinguishing Aβ-PET(+) from Aβ-PET(−) individuals across all cognitive stages. AUC = 0.964 (95% CI: 0.908–1). **(C)** ROC curves of LASSO logistic regression models of the two-gene model (*COL14A1* and *RUNX1T1*) distinguishing Aβ-PET(+) from Aβ-PET(−) individuals across all cognitive stages. AUC = 0.81 (95% CI: 0.561–1). **(D)** AUC for the LASSO model including *KCNMB2*, *NRG3*, and *HCN1* within the MCI and dementia subgroups. **(E)** ROC curve of the three-gene model (*KCNMB2, NRG3, and HCN1*) predicting Aβ-PET status among individuals with MCI or dementia. AUC = 0.97 (95% CI: 0.904–1). **(F)** ROC curves of LASSO logistic regression models of the three-gene model (*KCNMB2, NRG3, and HCN1*) predicting Aβ-PET status among individuals with MCI or dementia. AUC = 0.79 (95% CI: 0.568–1).

Figure 5A shows the LASSO coefficient profiles across the regularization path, and Figure 5B displays the ROC curve of the final two-gene model, which achieved an apparent AUC of 0.964 (95% CI: 0.908–1). Model performance was further assessed using leave-one-out cross-validation (LOOCV), yielding a more conservative AUC of 0.81 (95% CI: 0.561–1; Figure 5C). This model demonstrated moderate discriminatory ability to distinguish A*β*-PET(+) from Aβ-PET(−) individuals across cognitive stages (SCD, MCI, and dementia). Given that candidate genes were selected prior to LOOCV, these results should be interpreted as internally validated estimates. Therefore, *COL14A1* and *RUNX1T1* should be considered candidate blood-based biomarkers of Aβ pathology that require further validation in independent cohorts.

To examine the robustness of these associations, we performed an additional Firth penalized logistic regression adjusting for age and APOE ε4 status. As shown in Table 2, *RUNX1T1* remained significantly associated with Aβ status (β = −3.291, *p* = 0.004), while *COL14A1* showed a trend-level association (β = −1.828, *p* = 0.067). The overall model was statistically significant (likelihood ratio test χ^2^ = 21.15, df = 4, *p* = 2.96 × 10^−4^), supporting the presence of a multivariable association beyond the effects of major clinical covariates.

**Table 2:** Firth logistic regression analysis of Aβ status-associated variables.

Variable	Coefficient (β)	SE	95% CI	Chi-Square	*P* value
COL14A1	−1.828	1.045	[−8.446, 0.094]	3.355	0.067
RUNX1T1	−3.291	1.621	[−13.741, −0.714]	8.255	0.004
Age	0.050	0.075	[−0.126, 0.236]	0.382	0.537
APOE ε4 status	0.200	1.197	[−2.556, 4.222]	0.023	0.880

Model fitted by Firth’s Penalized Maximum Likelihood due to restricted sample size. The overall model significance was assessed using the likelihood ratio test (χ^2^ = 21.15, df = 4, P = 2.96 × 10^−4^).

To further explore gene signatures associated with disease progression, we constructed a second LASSO model, this time restricted to individuals in the MCI and dementia groups only. This model identified three genes: *KCNMB2*, *NRG3*, and *HCN1*, with coefficients of −0.023, 0.898, and 1.037, respectively.

Figure 5D presents the LASSO coefficient path, while Figure 5E shows the corresponding ROC curve, with an apparent AUC of 0.97 (95% CI: 0.904–1). In internal LOOCV evaluation, the model achieved an AUC of approximately 0.79 (95% CI: 0.568–1; Figure 5F), suggesting that the apparent model performance may be overestimated due to the limited sample size. This model still demonstrated reasonable discriminatory capacity in later disease stages. Among the selected genes, *NRG3* and *HCN1* showed strong positive associations, while *KCNMB2* exhibited a weaker yet consistent negative association.

To further explore the predictive power of the selected gene signatures in distinct clinical stages, we performed LASSO logistic regression separately in the MCI and dementia groups. Using LOOCV, the model for the MCI group (Supplementary Figure S5A) achieved an AUC of 0.91, while the dementia group model (Supplementary Figure S5B) yielded an AUC of 0.84. These findings suggest that the identified gene signatures retain discriminatory potential within specific disease stages, although independent validation will be required to confirm their generalizability.

### 3.9 Correlation analysis between blood gene expression and brain structure

**Figure 6:**
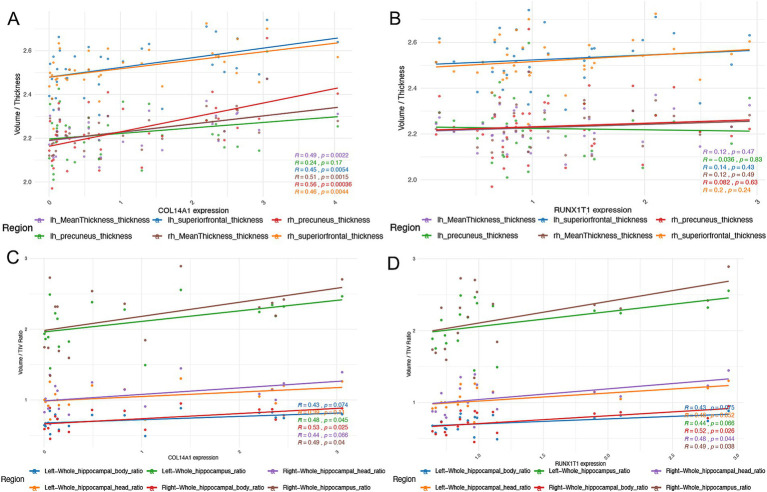
Correlation between PBMC gene expression and brain structural metrics. **(A)** *COL14A1* expression versus cortical thickness. **(B)** *RUNX1T1* expression versus cortical thickness. **(C)** *COL14A1* expression versus hippocampal subfield volumes. **(D)** *RUNX1T1* expression versus hippocampal subfield volumes. After quality control, a total of *n* = 20 participants were included.

Based on RNA-seq screening and qRT-PCR validation, *RUNX1T1* and *COL14A1* were consistently downregulated in Aβ-PET–positive individuals compared with Aβ-PET–negative counterparts (*p* < 0.001). To further explore the relationship between these genes and brain structure, correlation analyses were performed, excluding samples with poor or unreadable MRI data. *COL14A1* expression in PBMCs was significantly positively correlated with cortical thickness in multiple regions, including the superior frontal gyrus, precuneus, and global mean cortical thickness, suggesting a potential role in maintaining cortical structure (Figure 6A). In contrast, *RUNX1T1* showed no significant correlation with cortical thickness (Figure 6B). Further analysis indicated that *COL14A1* was positively associated with total hippocampal volume bilaterally, with the strongest correlation observed in the hippocampal body. Although the hippocampal head did not reach statistical significance, the trend was consistent (Figure 6C). Overall, *COL14A1* expression showed broad associations with both cortical and hippocampal structures, whereas *RUNX1T1* correlations were mainly restricted to hippocampal volume, particularly on the right side (Figure 6D).

## 4 Discussion

Aβ deposition is a central pathological hallmark of cognitive decline, and its early identification is crucial for timely intervention and disease modification. By integrating peripheral blood transcriptomics with MRI-derived structural metrics, this study explored molecular alterations associated with Aβ pathology across SCD, MCI, and dementia, providing insights into stage-specific molecular patterns associated with Aβ burden.

At the global transcriptomic level, Aβ accumulation was accompanied by widespread molecular dysregulation. The downregulation of ECM and cytoskeleton-related genes may compromise cell–matrix support and neurovascular unit integrity, whereas dysregulation of synaptic genes directly impairs neurotransmission and neuronal plasticity. Meanwhile, the upregulation of immune–endocrine and splicing/transcriptional pathways likely represents both compensatory and inflammation-driven responses (Griswold et al., 2020). This dynamic “injury–compensation–imbalance” pattern provides a conceptual framework for understanding stage-specific transcriptomic changes during AD progression.

When comparing hub genes and pathways across SCD, MCI, and dementia, we observed high similarity between SCD and MCI groups but marked divergence from the dementia group. The SCD/MCI stages were characterized by hub genes such as *IFIT1*, *IFIT3*, and *RSAD2*, which are involved in interferon and antiviral responses. These findings suggest that early Aβ accumulation is associated with activation of innate immune-related pathways in peripheral blood, which may reflect an early systemic response to pathological changes (Liu et al., 2024; Song et al., 2022). However, given the cross-sectional nature of the data and the absence of clear cognitive impairment differences in the SCD subgroup, the functional interpretation of these immune alterations remains speculative. In contrast, the dementia stage exhibited hub genes enriched in synaptic transmission and glutamatergic signaling (e.g., *GRIA1/2*, *GRIN2B*, and *CACNG3*). These genes are functionally related to synaptic activity, suggesting that later disease stages are accompanied by transcriptional changes associated with neural system–related processes (Korinek et al., 2024). Immune and extracellular matrix–related alterations appeared to be more prominent in the SCD and MCI groups, whereas the dementia group exhibited broader transcriptomic changes involving pathways related to neural function. These observations suggest the presence of stage-associated differences in peripheral gene expression across clinical groups. Accordingly, peripheral transcriptomic changes may represent candidate molecular signatures associated with different clinical stages and warrant further stage-specific investigation.

Among the identified hub genes, *COL14A1* and *RUNX1T1* emerged as promising candidate peripheral markers, as both showed concordant downregulation across cognitive stages and demonstrated discriminatory potential for distinguishing Aβ-PET(+) from Aβ-PET(−) individuals. Beyond their diagnostic value, the biological functions of these genes provide important mechanistic clues linking peripheral molecular alterations to central neurodegeneration. Biologically, *COL14A1* encodes a collagen-associated ECM component involved in structural organization and cell–matrix signaling (Morris et al., 2010). In the central nervous system, ECM integrity is essential not only for structural support but also for maintaining vascular basement membrane stability and neurovascular unit (NVU) function (De Luca et al., 2020). Increasing evidence indicates that ECM remodeling plays a critical role in preserving blood–brain barrier (BBB) integrity by regulating endothelial–pericyte interactions and vascular permeability (Hawkins and Davis, 2005). In this context, the observed downregulation of *COL14A1* may reflect a state of compromised ECM homeostasis, potentially weakening vascular–neuronal support systems and predisposing the brain to BBB dysfunction.

Notably, BBB breakdown and NVU impairment have been increasingly recognized as early events in AD pathogenesis, preceding overt neurodegeneration and accelerating Aβ accumulation by reducing clearance efficiency and amplifying neuroinflammatory signaling. The observed correlations between *COL14A1* expression and cortical thickness as well as hippocampal volume suggest a potential association between peripheral transcriptomic alterations and structural brain measures. Given the limited sample size, these findings should be considered exploratory and require validation in larger cohorts. We therefore speculate that peripheral downregulation of *COL14A1* may serve as a molecular indicator of systemic ECM remodeling that parallels, or potentially contributes to, neurovascular dysfunction, ultimately facilitating cortical and hippocampal atrophy in Aβ-positive individuals.

*RUNX1T1*, in contrast, represents a distinct but complementary mechanistic axis. As a transcriptional regulator implicated in neurodevelopment and neuronal differentiation, *RUNX1T1* plays an important role in maintaining stable transcriptional programs required for neuronal integrity (Bergmann et al., 2022). Its consistent downregulation across disease stages, together with region-specific associations with hippocampal volume (Linqing et al., 2015; Zhang et al., 2009), suggests that *RUNX1T1* may contribute to Aβ-related neurodegeneration through disruption of gene regulatory networks rather than structural matrix alterations alone. Given the heightened vulnerability of hippocampal circuits to metabolic stress and synaptic dysfunction, reduced RUNX1T1 expression may exacerbate transcriptional instability, impair neuronal maintenance pathways, and lower the resilience of hippocampal networks to Aβ-induced injury.

In addition, several genes, including *HCN1*, *NRG3*, and *KCNMB2*, were differentially expressed in the MCI and dementia groups, highlighting their potential relevance to molecular changes associated with later-stage Aβ pathology. Experimental and genetic studies have shown that *HCN1* deletion increases Aβ accumulation (Zhang et al., 2024; Saito et al., 2012), while *NRG3* and *KCNMB2* variants are linked to hippocampal pathology and the age at onset of AD (Wang et al., 2014; Katsumata et al., 2017). These findings support their involvement in advancing neuropathology and clinical deterioration.

Several limitations should be acknowledged. No formal *a priori* power analysis was performed. Although the cohort size is comparable to previous exploratory transcriptomic studies, the limited sample size may have reduced statistical power and increased the risk of both false-negative and unstable effect estimates. The sample size was relatively small and demographically homogeneous, which may limit generalizability. Given the relatively small sample size, differential expression results should be interpreted cautiously despite the application of Benjamini–Hochberg FDR correction, as residual false-positive findings and unstable effect estimates cannot be completely excluded. Moreover, the cross-sectional design and correlative nature of blood expression–MRI associations preclude causal inference. Future studies should include larger, multi-center, and multi-ethnic cohorts, along with longitudinal follow-up to delineate temporal expression dynamics. Functional investigations using cellular and animal models are warranted to elucidate the roles of *RUNX1T1*, *COL14A1*, and other candidate genes—through gain- and loss-of-function approaches—to assess their effects on ECM remodeling, vascular integrity, synaptic function, and Aβ metabolism. Integrative multi-omics approaches, including proteomics, metabolomics, and epigenomics, may further clarify the molecular hierarchy and regulatory networks underlying early Aβ-driven pathogenesis. Although major demographic variables were balanced across groups, residual confounding effects from unmeasured factors cannot be completely excluded. In addition, the lack of direct immune cell composition measurement in PBMC samples is a limitation. Although xCell analysis showed no significant group differences, residual confounding from cellular heterogeneity cannot be excluded. Candidate genes were selected using the full dataset prior to LOOCV analysis. Therefore, the reported predictive performance should be interpreted as an internally validated estimate and requires confirmation in independent external cohorts.

In summary, by integrating blood transcriptomic profiles with neuroimaging data across cognitive stages, this study delineates stage-specific molecular signatures associated with Aβ pathology. The findings identify *RUNX1T1* and *COL14A1* as promising peripheral biomarkers and mechanistic targets for future translational and experimental studies.

## Data Availability

The raw sequence data generated in this study have been deposited in the Genome Sequence Archive (Genomics, Proteomics & Bioinformatics 2025) in the National Genomics Data Center (Nucleic Acids Research 2025), China National Center for Bioinformation/Beijing Institute of Genomics, Chinese Academy of Sciences (GSA Human: HRA019152). The data are publicly accessible at https://ngdc.cncb.ac.cn/gsa-human/.

## Footnotes

1
http://surfer.nmr.mgh.harvard.edu

## Glossary

AD: Alzheimer’s Disease
Aβ: Amyloid Beta
Aβ-PET: Amyloid Beta Positron Emission Tomography
CSF: Cerebrospinal Fluid
PBMCs: Peripheral Blood Mononuclear Cells
SCD: Subjective Cognitive Decline
MCI: Mild Cognitive Impairment
MMSE: Mini-Mental State Examination
MoCA-BC: Montreal Cognitive Assessment-Basic
AVLT: Auditory Verbal Learning Test
AFT: Animal Fluency Test
BNT: Boston Naming Test
STT: Shape Trail Test
SUVR: Standardized Uptake Value Ratio
GO: Gene Ontology
KEGG: Kyoto Encyclopedia of Genes and Genomes
RRA: Robust Rank Aggregation
PPI: Protein–Protein Interaction
MCC: Maximal Clique Centrality
STRING: Search Tool for the Retrieval of Interacting Genes/Proteins
LASSO: Least Absolute Shrinkage and Selection Operator
LOOCV: Leave-One-Out Cross-Validation
qRT-PCR: Quantitative Reverse Transcription Polymerase Chain Reaction
ROC: Receiver Operating Characteristic
AUC: Area Under the Curve
SEM: Standard Error of the Mean
NfL: Neurofilament Light Chain
GFAP: Glial Fibrillary Acidic Protein
NVU: Neurovascular Unit
BBB: Blood–Brain Barrier
