# Supplementary material for: Blood transcriptomic signatures link β-amyloid deposition to molecular pathways across SCD, MCI, and dementia
Source: Front Aging Neurosci. 2026 Jul 15;18:1816733. doi: 10.3389/fnagi.2026.1816733 (PMC13416258; doi:10.3389/fnagi.2026.1816733)
Supplement: Supplementary file 1 [file Data_Sheet_1.DOCX]

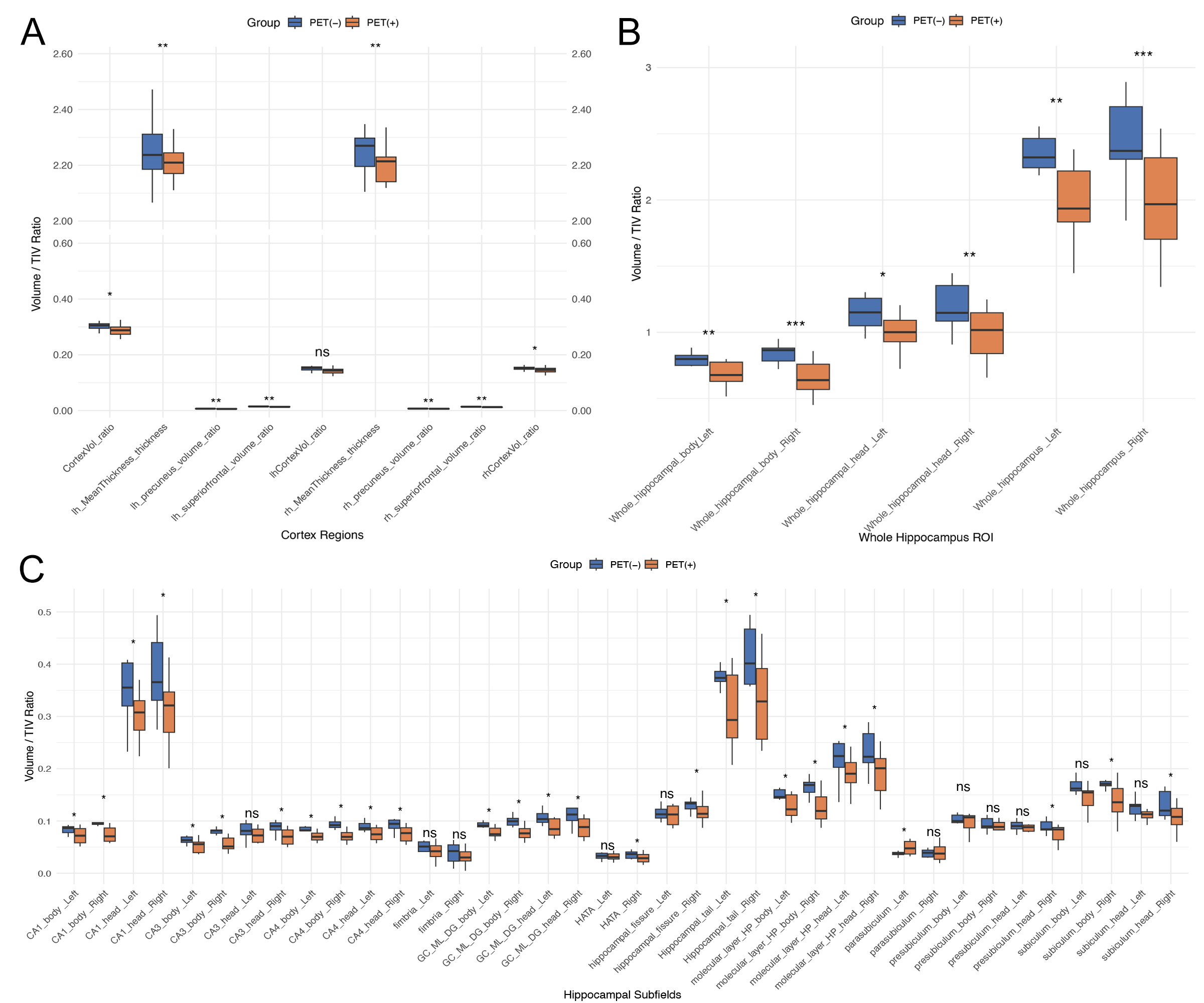


**Figure S1. Brain structural metrics in Aβ-PET(+) and Aβ-PET(−) individuals.**

1. Cortical metrics; (B–C) Hippocampal metrics. Statistical comparisons between groups were performed using two-sided t-tests, and p values were adjusted for multiple comparisons using the Benjamini–Hochberg false discovery rate (FDR) procedure. Significance is indicated based on adjusted P values: *P < 0.05, **P < 0.01, ***P < 0.001, ****P < 0.0001. ns, not significant.


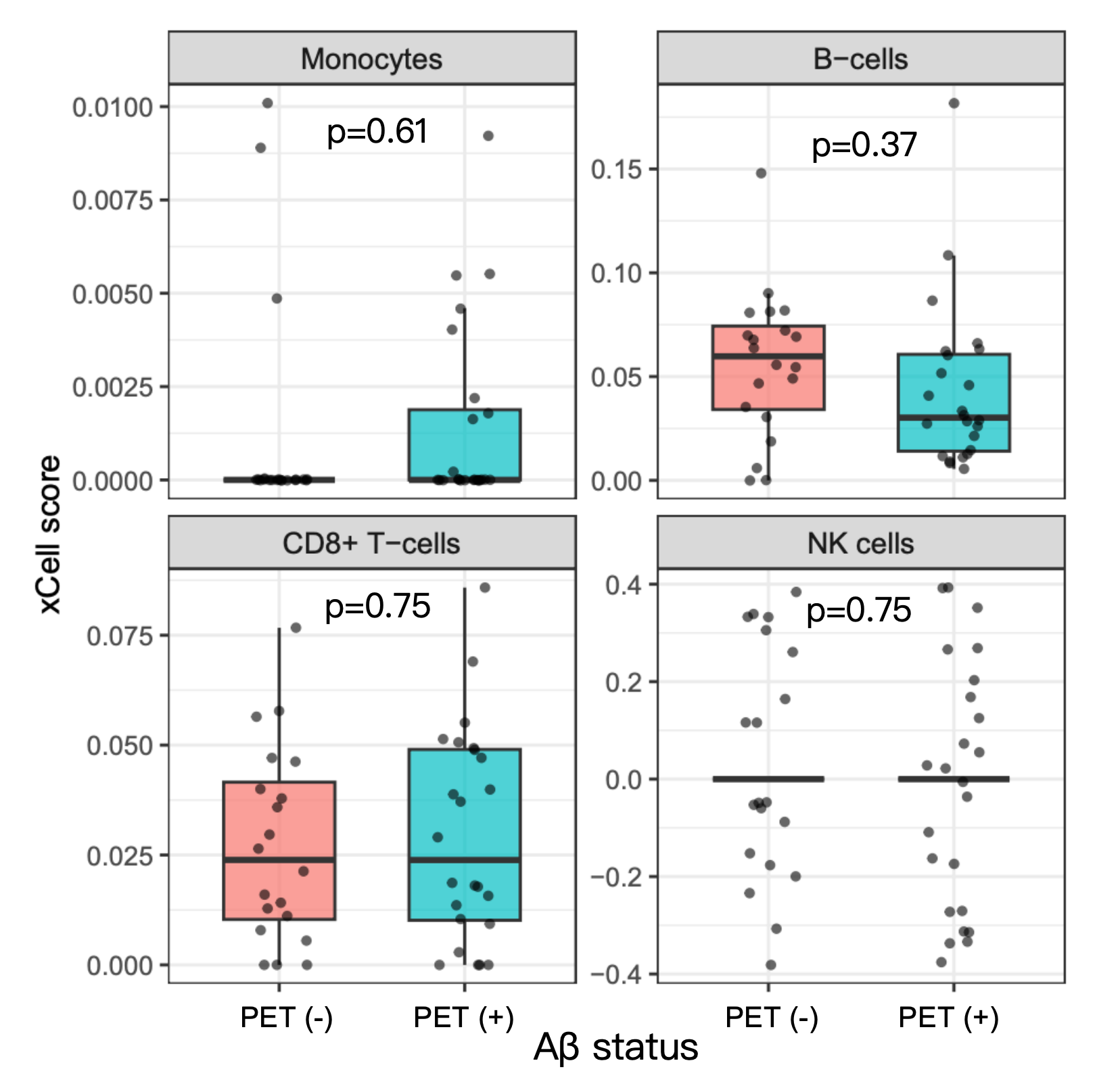


**Figure S2. Immune cell composition deconvolution analysis using xCell.**
xCell algorithm was applied to estimate relative abundances of immune cell populations in PBMC transcriptomic data. Major immune cell types, including monocytes, B cells, CD8+ T cells, and NK cells, showed no significant differences between Aβ-PET(+) and Aβ-PET(−) groups after multiple testing correction (Wilcoxon rank-sum test, all adjusted P > 0.05), indicating comparable immune cell composition across groups.


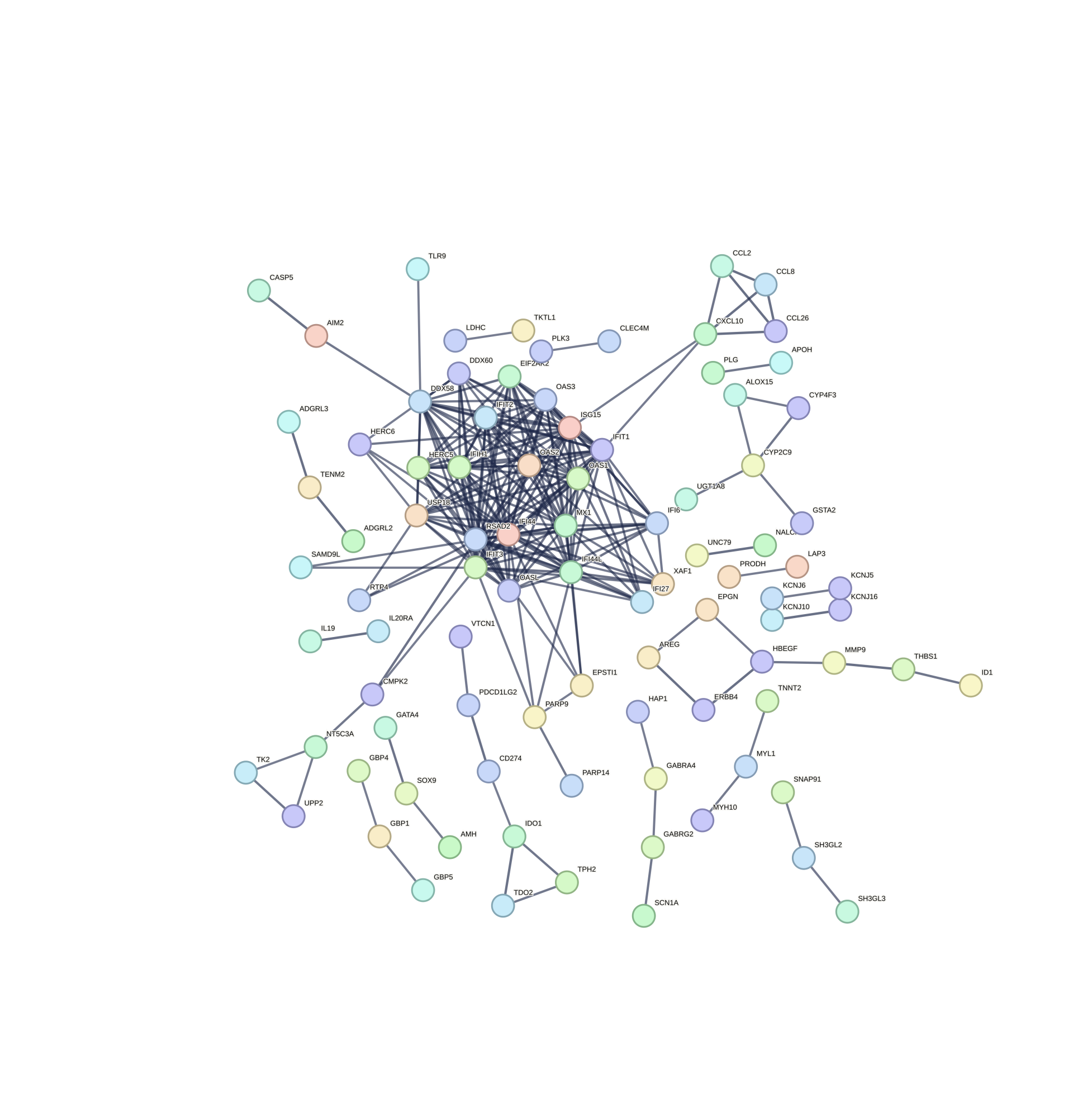


A


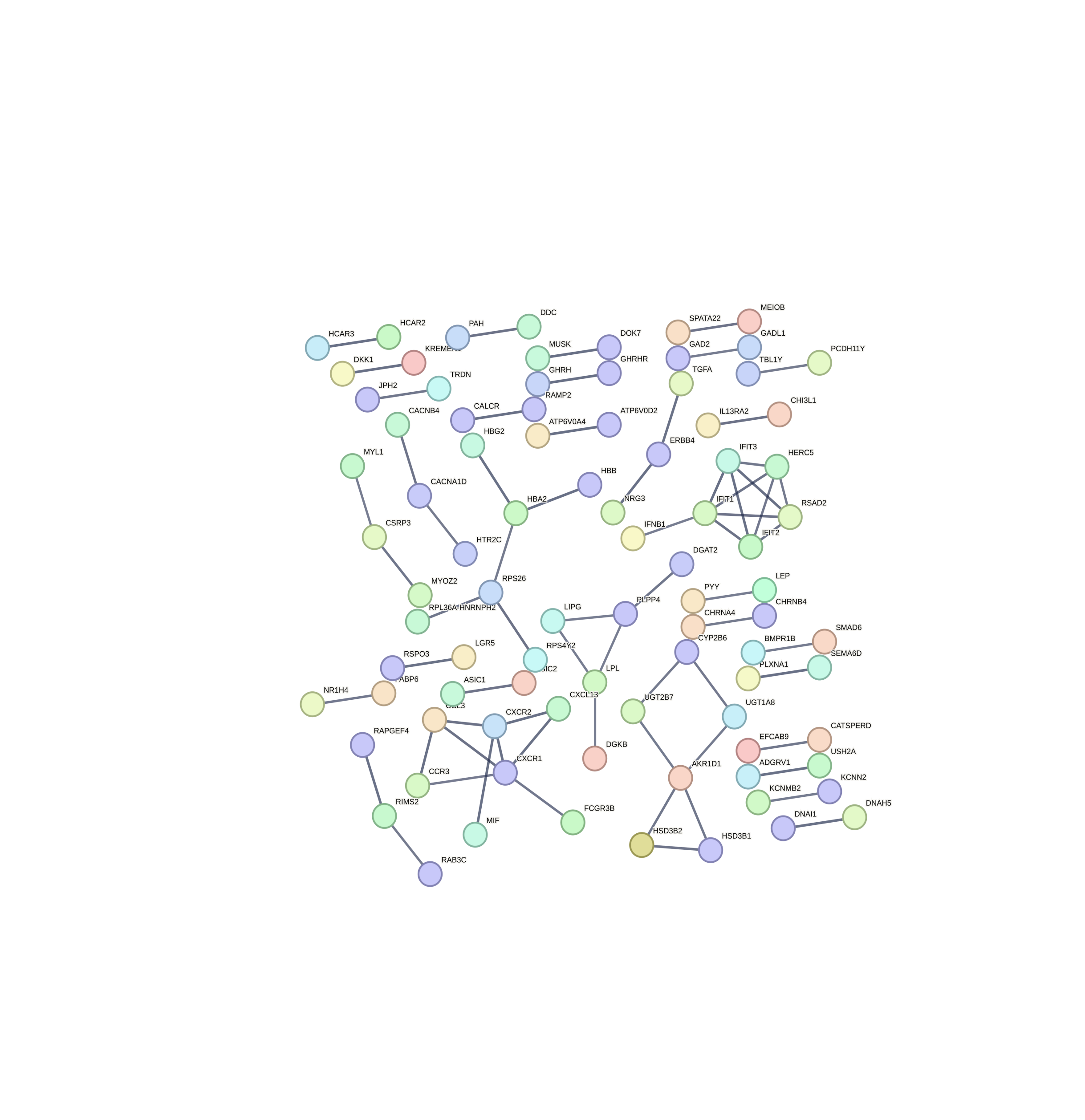


B


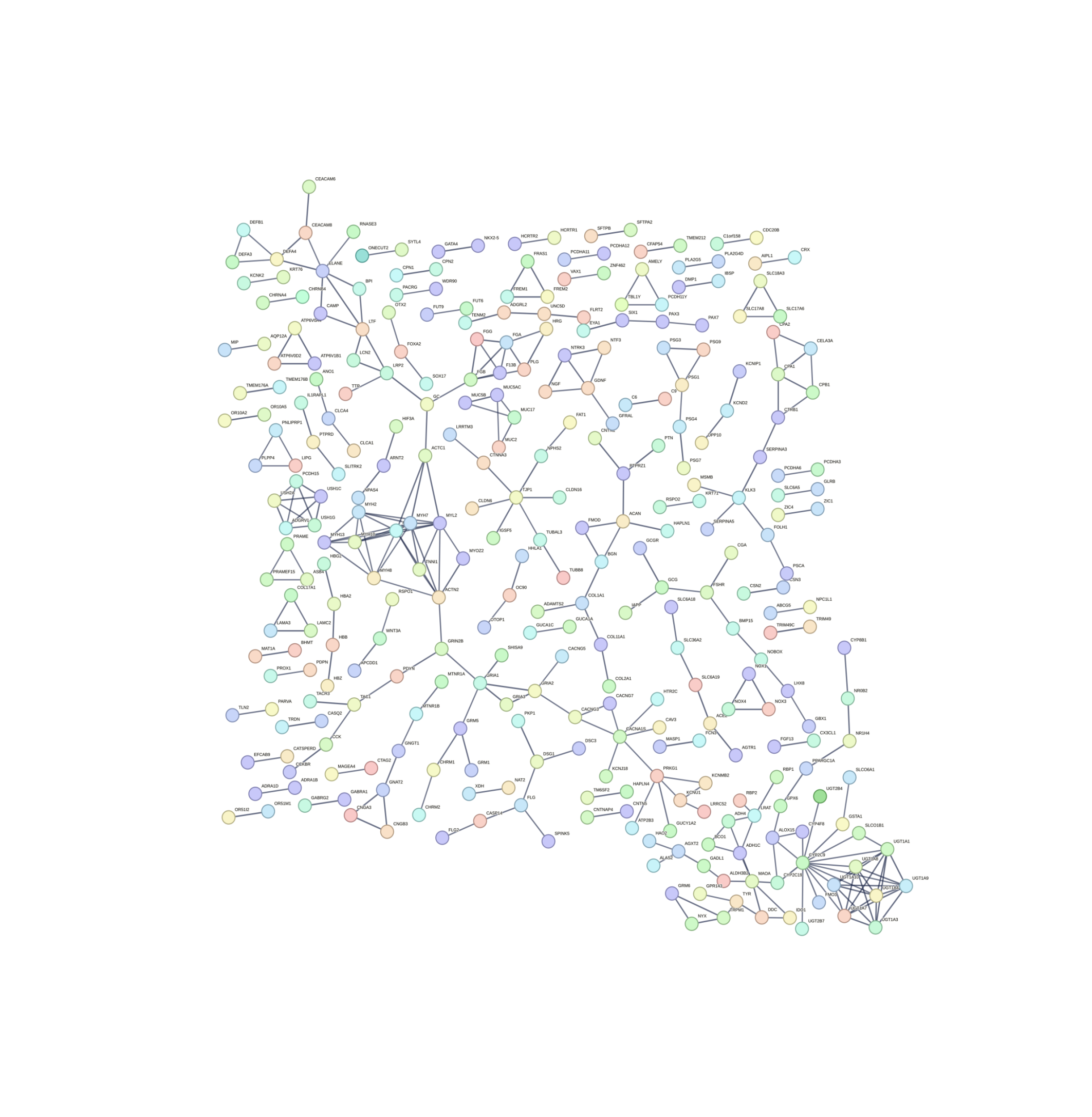


C

**Figure S3. STRING Network Analysis of Differentially Expressed Genes between Aβ-PET(+) and Aβ-PET(-)**

(A) CN group; (B) MCI group; (C) Dementia group; High Confidence Interactions, Minimum Required Interaction Score: 0.9


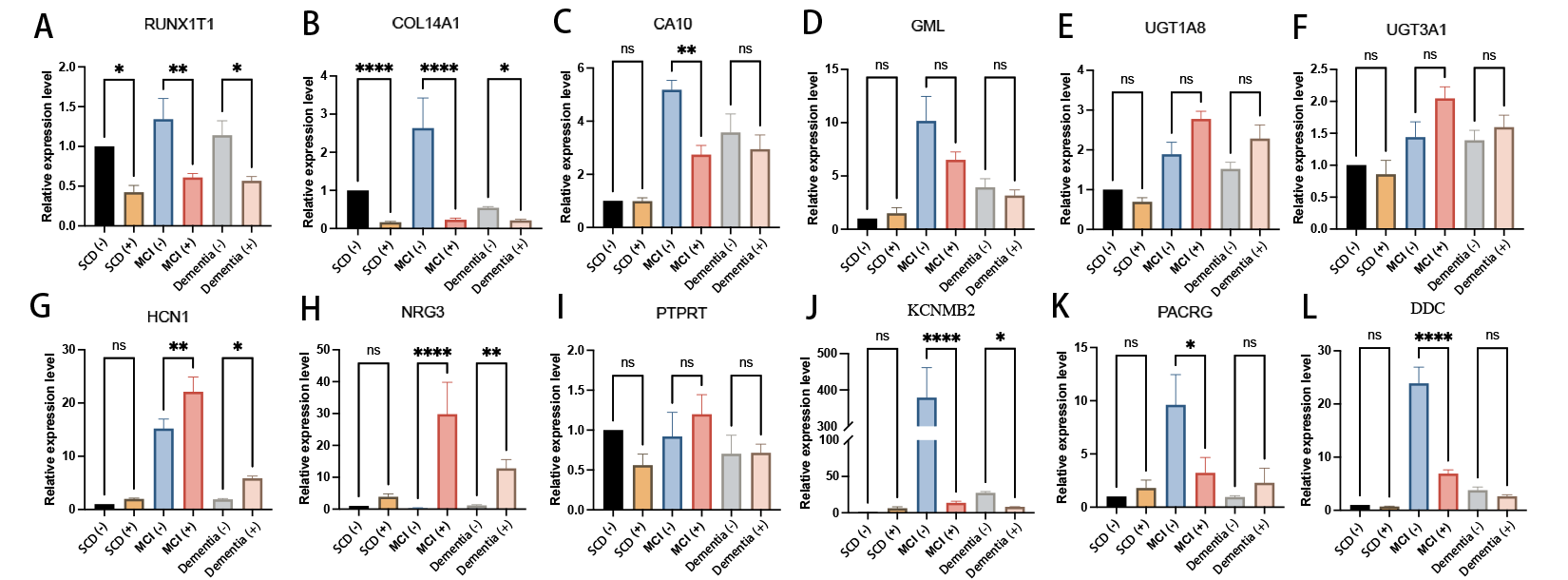


**Figure S4. qRT-PCR validation of DEGs**

Data are presented as mean ± SEM, n = 8. * p-value < 0.05, ** p-value < 0.01, *** p-value < 0.001, and **** p-value <0.0001. ns, No significance.


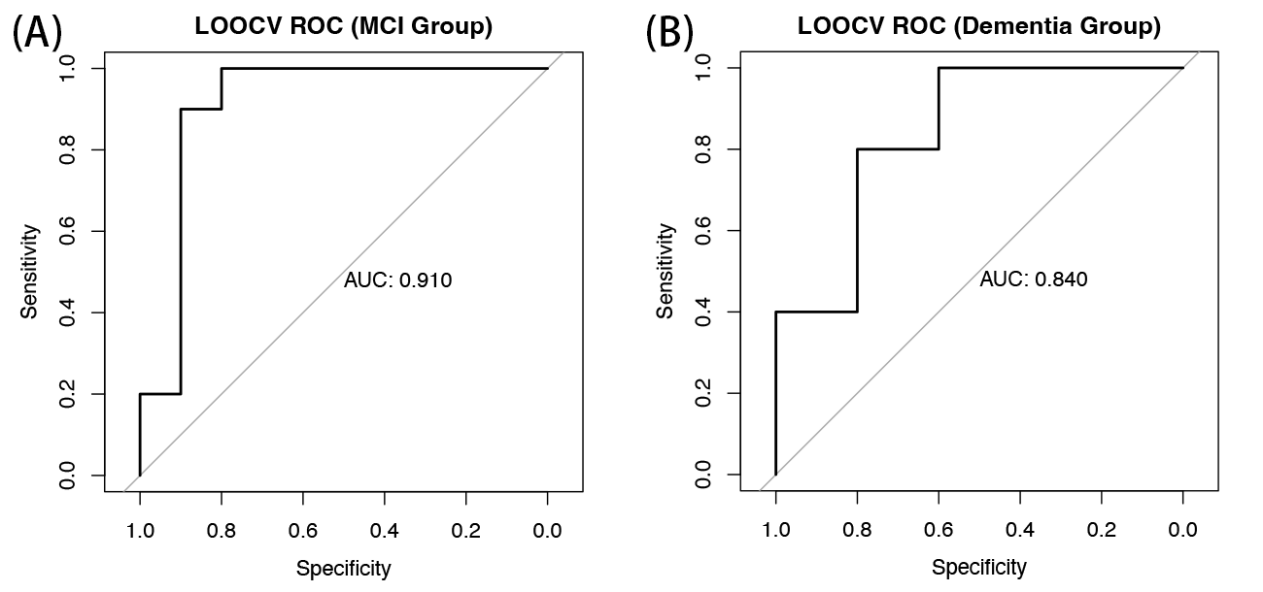


**Figure S5. Stage-Specific Predictive Models for Aβ-PET Status Based on Candidate Biomarker Genes**

ROC curves of LASSO logistic regression models using a panel of candidate biomarker genes (*RUNX1T1*, *COL14A1*, *KCNMB2*, *NRG3*, and *HCN1*) to predict Aβ-PET status in (A) the MCI group and (B) the dementia group. Models were trained with leave-one-out cross-validation (LOOCV).
