# Supplementary material for: Blood transcriptomic signatures link β-amyloid deposition to molecular pathways across SCD, MCI, and dementia
Source: Front Aging Neurosci. 2026 Jul 15;18:1816733. doi: 10.3389/fnagi.2026.1816733 (PMC13416258; doi:10.3389/fnagi.2026.1816733)
Supplement: Supplementary file 2 [file Supplementary_file_1.DOCX]

| **Gene** | **Sequence 5'--> 3'** | **Length** |
| --- | --- | --- |
| h-KCNMB2-F | GAGGACCGAGCTATTCTCCTG | 21 |
| h-KCNMB2-R | TGTTTCCGTGATGGACGCATT | 21 |
| h-DDC-F | ATTCATCTGCCCTGAGTTCCG | 21 |
| h-DDC-R | CCAATAGCCATTTGTGGGGAT | 21 |
| h-PACRG-F | AAATGCCCAGACAAGATGCC | 20 |
| h-PACRG-R | GGCCTCTCACGACTGAGTT | 19 |
| h-HCN1-F | TACTGCCAGTGTTCGAGCTG | 20 |
| h-HCN1-R | AGTCGGTCAATGGCAACTGT | 20 |
| h-NRG3-F | ACGACGACATATTCCACAGAGC | 22 |
| h-NRG3-R | CCGGTCAGGGTTTCGATCAC | 20 |
| h-PTPRT-F | TCTGCGACTCCAAAACGTGG | 20 |
| h-PTPRT-R | ACAGCGGTACTTGCTGACG | 19 |
| h-COL14A1-F | TTCAGACTGGTTCGGCATTTC | 21 |
| h-COL14A1-R | CTGTGCAAGACCAATTCGTGT | 21 |
| h-RUNX1T1-F | ATGCCAGACTCACCTGTGGAT | 21 |
| h-RUNX1T1-R | GGCTGTAGGAGAATGGCTCG | 20 |
| h-UGT1A8-F | TTGATGCCTGTGCGTTAATTGT | 22 |
| h-UGT1A8-R | GGCAACCTATTCCCCTGGC | 19 |
| h-GML-F | TGAGATGCCATGACTGTGCG | 20 |
| h-GML-R | GGAGATTGTCATACAGCGCCT | 21 |
| h-UGT3A1-F | CAAGGAGCATTTCCCAGAAGG | 21 |
| h-UGT3A1-R | TCTCAGTGGTTCAGGATACACTC | 23 |
| h-CA10-F | CTTTCTGGGGATTGGTGAACTC | 22 |
| h-CA10-R | TGTGACTGGTCTCTATGTTGACT | 23 |

Table S1. Primer sequences used for qRT-PCR

Note: qRT-PCR: quantitative real-time PCR; This table lists the forward and reverse primer sequences used for the quantification of target gene mRNA expression.
